# Future treatments for hereditary hemorrhagic telangiectasia

**DOI:** 10.1186/s13023-019-1281-4

**Published:** 2020-01-07

**Authors:** Florian Robert, Agnès Desroches-Castan, Sabine Bailly, Sophie Dupuis-Girod, Jean-Jacques Feige

**Affiliations:** 1 Univ. Grenoble Alpes, Inserm, CEA, Laboratory Biology of Cancer and Infection, F-38000 Grenoble, France; 2 Hospices Civils de Lyon, Service de Génétique, Hôpital Femme-Mère-Enfants, F-69677 Bron, France; 3 Centre National de Référence pour la Maladie de Rendu-Osler, F-69677 Bron, France

**Keywords:** Hereditary hemorrhagic telangiectasia, Vascular malformations, Bone morphogenetic protein signaling, Drug repositioning, Bevacizumab, Tacrolimus, ALK1, High throughput screening

## Abstract

*Hereditary Hemorrhagic Telangiectasia* (HHT), also known as Rendu-Osler syndrome, is a genetic vascular disorder affecting 1 in 5000–8000 individuals worldwide. This rare disease is characterized by various vascular defects including epistaxis, blood vessel dilations (telangiectasia) and arteriovenous malformations (AVM) in several organs. About 90% of the cases are associated with heterozygous mutations of *ACVRL1* or *ENG* genes, that respectively encode a bone morphogenetic protein receptor (activin receptor-like kinase 1, ALK1) and a co-receptor named endoglin. Less frequent mutations found in the remaining 10% of patients also affect the gene *SMAD4* which is part of the transcriptional complex directly activated by this pathway. Presently, the therapeutic treatments for HHT are intended to reduce the symptoms of the disease. However, recent progress has been made using drugs that target VEGF (vascular endothelial growth factor) and the angiogenic pathway with the use of bevacizumab (anti-VEGF antibody). Furthermore, several exciting high-throughput screenings and preclinical studies have identified new molecular targets directly related to the signaling pathways affected in the disease. These include FKBP12, PI3-kinase and angiopoietin-2. This review aims at reporting these recent developments that should soon allow a better care of HHT patients.

## Background

*Hereditary Hemorrhagic Telangiectasia* (HHT), also known as Rendu-Osler syndrome, is a genetic vascular disorder affecting 1 in 5000–8000 individuals worldwide, with regional differences and higher prevalence areas associated with founder effects [1–4]. This rare disease (ORPHA774; https://www.orpha.net/consor/cgi-bin/index.php?lng=EN) is characterized by various vascular defects including epistaxis, blood vessel dilations (telangiectasia) and arteriovenous malformations (AVM) in lungs, liver and brain. Epistaxis is the most frequent clinical manifestation of HHT, affecting more than 95% of patients [5]. Pulmonary AVMs are observed in 15–45% of patients but remain frequently undiagnosed and asymptomatic. Hepatic AVMS are observed in more than 70% of patients depending on the screening technique used but only 8% of patients will develop symptomatic liver disease [6]. Gastrointestinal telangiectasias are quite frequent (70% of patients) and may lead to hemorrhages and anemia [7]. Cerebral AVMs are less frequent (10–23% of HHT patients) but their consequences may be fatal.

90% of the HHT cases are associated with heterozygous mutations of *ACVRL1* or *ENG* genes, that respectively encode a bone morphogenetic protein receptor (activin receptor-like kinase 1, ALK1) and a co-receptor named endoglin. Less frequent mutations found in the remaining 10% of patients also affect genes that encode components of the BMP9/ALK1 signaling pathway. Presently, the therapeutic treatments for HHT are intended to reduce the symptoms of the disease. However, no mechanism-based targeted therapy is available so far. In this review, we will focus on the development of new drugs aiming at correcting the altered signaling pathways in HHT patients. These include drugs that target VEGF (vascular endothelial growth factor) and the angiogenic pathway as well as repositioned drugs identified by high throughput screening strategies.

## Main text

### Genetic and mechanistic presentation of HHT

HHT is an autosomal dominant genetic disease that commonly results from monoallelic mutations in either *ENG* (HHT1, OMIM #187300) or *ACVRL1* (HHT2, OMIM #600376) genes [8, 9]. *ACVRL1* encodes the BMP (Bone Morphogenetic Protein) receptor ALK1 (activin receptor-like kinase 1) whose expression is grossly restricted to the vascular and lymphatic endothelia [10, 11]. Endoglin (encoded by *ENG*) is also an endothelial-specific receptor for BMPs which is devoid of intracellular kinase activity and acts as a co-receptor in complex with ALK1 [12, 13]. Mutations of either one of these two genes are observed in 90% of the genetically screened patients. In addition, mutations in *SMAD4* (encoding the transcription factor Smad4) have been described in a subset of HHT patients which present a juvenile polyposis/HHT overlap syndrome (JP-HHT, OMIM #175050) but the frequency of these mutations does not exceed 2% of the HHT patient population [14–16]. More recently, mutations in the *GDF2* gene (encoding BMP9) have been described in a vascular anomaly syndrome with phenotypic overlap with HHT (HHT5, OMIM #615506), but the contribution of *GDF2* mutations to HHT is estimated to be much less than 1% [17, 18].

**Fig. 1:**
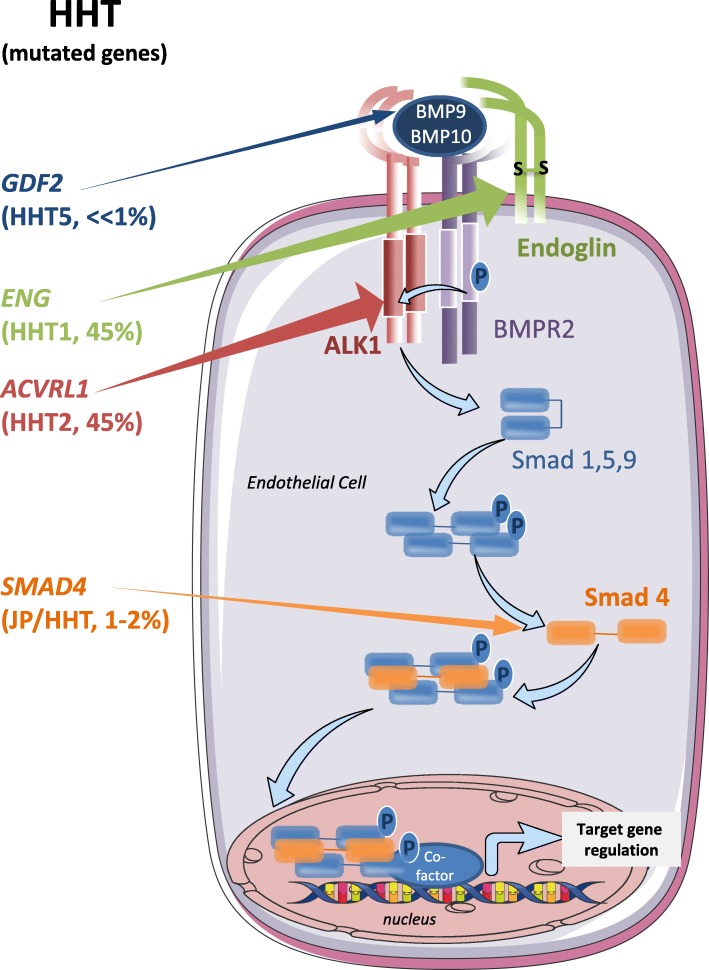
Mutated genes in HHT encode members of the BMP9/BMP10 signaling pathway. The cartoon depicts the BMP9/BMP10 signaling pathway in endothelial cells. After ligand binding to cell surface receptors, signal transduction proceeds through phosphorylation of the type 1 receptor ALK1, phosphorylation of Smad 1/5/9, translocation of the Smad complex to the nucleus and transcriptional effects on target genes, as indicated by blue arrows. The left part of the Figure lists the names of the genes that are mutated in HHT patients and the arrows point to their gene products. The frequency of the mutations is indicated in % between the parentheses

It is exciting to observe that the products of these 4 mutated genes all belong to the same signaling pathway (Fig. 1). Homodimeric BMP9 and BMP10, as well as the recently characterized BMP9-BMP10 heterodimer, are high-affinity ligands of a receptor complex comprising ALK1, endoglin and a BMP type II receptor (BMPRII or ACTRIIA or ACTRIIB) [19–21]. Under activation by BMP9/10, this receptor complex phosphorylates the transcription factors Smad1, Smad5 or Smad9. Dimers of phospho-Smad1, phospho-Smad5 or phospho-Smad9 associate in a trimeric complex with Smad4 and translocate into the endothelial cell nucleus where they bind to BMP-responsive elements on the promoters of target genes and either enhance or repress their expression [22, 23]. HHT is thus now considered as a disease of the BMP9/10 pathway rather than a disease of the TGFß pathway, as initially thought [24].

About 550 distinct pathogenic mutations of *ACVRL1* and 490 pathogenic mutations of *ENG* have been reported in humans. They are registered in the ARUP database (http://arup.utah.edu/database/hht/). Mutations have been observed in all exons of both genes as well as in some intronic regions. Pathogenic mutations in the 5′-UTR (5′-untranslated region) of the *ENG* gene have also been reported [25]. Missense mutations and genetic deletions are the most common types of mutations observed in both *ACVRL1* and *ENG*. Functional analysis of a series of 19 *ACVRL1* mutations in HHT2*,* distributed in regions coding the extracellular domain, the GS box (glycine/serine-rich box) or the serine/threonine kinase domain of the receptor, revealed that almost all mutants were expressed at the cell surface but were unable to activate Smad1/5/9 phosphorylation and BMP-responsive reporter gene expression [26]. In addition, none of these mutants was able to act a dominant-negative repressor of wild-type receptor activity, indicating that HHT2 mutations trigger functional haploinsufficiency of BMP9 signaling [26]. In contrast, *ENG* mutations in HHT1 induce protein loss-of-function through dist**i**nct mechanisms [27]. Some endoglin mutants are unable to reach the plasma membrane during their biosynthesis and remain retained intracellularly. When retained in the endoplasmic reticulum, some mutants can dimerize with wild-type endoglin and impair its cell surface expression, acting as dominant-negative receptors, while other mutants cannot. Some mutants get normally expressed at the cell surface but are inactive such as mutants S278P and F282 V that are unable to bind BMP9 [27].

Mutations in *SMAD4* are detected in 1 to 2% suspected HHT clinical cases and are also frequently observed in the syndrome of juvenile polyposis (JP) and the mixed syndrome of HHT/JP [15, 28, 29]. The *SMAD4* mutations identified in JP-HHT patients are distributed throughout the gene and include nonsense, missense, frameshift, splice site mutations as well as partial or entire gene deletions, consistent with the inheritance of a loss-of-function allele [28, 29].

In 2013, the team of Pinar Bayrak-Toydemir identified *GDF2* gene mutations (encoding BMP9) in 3 unrelated HHT patients who had previously been tested negative for *ACVRL1*, *ENG* or *SMAD4* mutations [18]. These observations remained isolated and it is now admitted that these mutations are extremely rare in HHT.

As a summary, Fig. 1 presents the different mutations observed in HHT, that all affect components of the BMP9/BMP10 signaling pathway. All these mutations are loss-of-function mutations.

As the clinical penetrance of the disease is highly variable, even within members of the same family bearing the same mutation, and since the vascular defects preferentially concern certain vascular beds (liver, lungs, brain) and develop in localized regions of the affected organs, it has long been postulated that a second local hit might be required to initiate the pathological process. On animal models of HHT, Paul Oh has elegantly shown that a local injury (e.g. burn wounding of a mouse ear) inflicted on *Alk1*-deficient mice triggers an abnormal revascularization (with dilated and tortuous vessels resembling HHT arteriovenous malformations) [30]. Increased tissue perfusion provoked by a local VEGF (vascular endothelial growth factor) surge has also been reported to trigger capillary dysplasia in the brain of *Alk1*^*+/−*^ heterozygous mice [31]. Even more interestingly, Doug Marchuk’s team has recently identified low-frequency somatic mutations in 9/19 human telangiectasias analyzed by deep sequencing and has confirmed on 7 samples that the germline and somatic mutations exist in trans configuration, resulting in a biallelic loss of either the *ENG* or the *ACVRL1* gene [32]. This would suggest that the second hit could be a somatic genetic mutation.

**Fig. 2:**
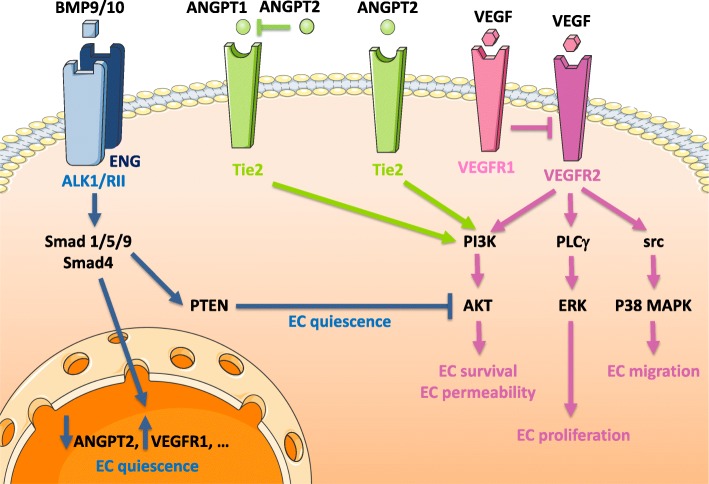
BMP9 and BMP10 induce vascular quiescence by various mechanisms. Through ALK1 phosphorylation of Smad1/5/9, BMP9 or BMP10 triggers transcriptional effects that induce vascular quiescence, including repression of ANGPT2 (angiopoietin 2) and induction of VEGFR1 expressions. In parallel, BMP9 inhibits the phosphorylation of the phosphatase PTEN (which is active in its unphosphorylated form), thereby inhibiting the activity of PI3K, a downstream effector of both VEGF and ANGPT2. ANGPT2 signaling is complex: when ANGPT1 (angiopoietin 1) is present, ANGPT2 acts as an antagonist of ANGPT1 and prevents the phosphorylation of the Tie2 receptor and the activation of PI3K. When ANGPT2 is present in large excess over ANGPT1, it acts as an agonist of the Tie2 receptor and stimulates PI3 Kinase. Tie2 activation is pro-angiogenic. VEGF activates different signaling pathways (PI3K/AKT, PLCγ/ERK, src/p38MAPK) which trigger a variety of biological responses (EC (endothelial cell) survival, permeability, proliferation and migration). VEGFR1, whose expression is increased by BMP9, acts as a decoy VEGF receptor, thereby shutting down the pro-angiogenic VEGF signaling mediated by VEGFR2 Altogether, BMP9 and BMP10 maintain vascular quiescence by shutting down the pro-angiogenic VEGF and ANGPT2 signaling pathways.

In adults, BMP9 and BMP10 are mainly produced by the liver and the right cardiac atria, respectively. They are present in the blood circulation under both homodimeric and heterodimeric forms [21, 33]. All forms induce either stimulation or repression of target gene expression in a phospho-Smad-dependent manner but can also induce non-Smad signaling via p38 MAP Kinase, ERK or JNK [22]. ALK1 can also cross-talk with the VEGF, angiopoietin 2, Notch and Hippo pathways [22]. The main biological output of BMP9/10 signaling is the induction of vascular quiescence [34, 35]. Vascular endothelial cells are under constant influence of pro- and anti-angiogenic factors and the dysregulation of this balance triggers either active angiogenesis or vascular quiescence. As shown in Fig. 2, BMP9 effects on endothelial cells appear to be mediated by a combination of diverse mechanisms. On one hand, BMP9 activates the endothelial cell expression of VEGFR1, a high-affinity non-signaling receptor that serves as a decoy receptor and down-regulates the pro-angiogenic action of VEGF through its signaling receptor VEGFR2 [36, 37]. In parallel, BMP9 represses the endothelial expression of ANGPT2 (angiopoietin-2), another pro-angiogenic growth factor that acts via the tyrosine kinase receptor Tie2 [38, 39]. BMP9 down-regulates also both the expression and the phosphorylation of PTEN (Phosphatase and TENsin homolog), leading in turn to increased PTEN activity and decreased activity of PI3K (phosphatidylinositol-4,5 bisphosphate 3-kinase) and AKT, two key components of the VEGF and ANGPT2 signaling pathways [40–43].

### Future treatments for HHT

**Fig. 3:**
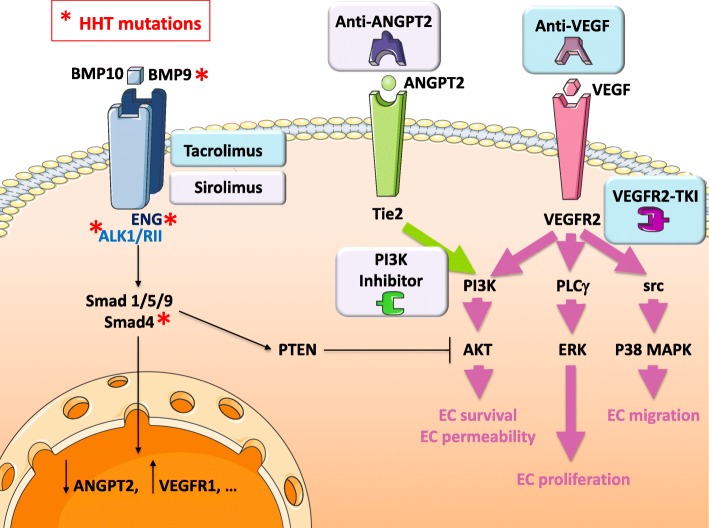
Future treatments for HHT. The HHT-causing mutations of genes encoding components of the BMP9/BMP10 signaling pathway (indicated by red asterisks), result in decreased downstream signaling (indicated by thinner arrows than in Fig. 2) and increased activity of the VEGF and ANGPT2 signaling pathways (indicated by thicker arrows than in Fig. 2). Several drugs that target these pathways are already in use in clinical trials (blue boxes) or under evaluation in preclinical studies (parma boxes) for HHT treatment. Currently evaluated HHT treatments target VEGF via anti-VEGF antibodies (bevacizumab) or VEGFR2 tyrosine kinase inhibitors (VEGFR2-TKI such as pazopanib). Tacrolimus and sirolimus were identified through recent high-throughput screening of FDA-approved drugs as activators of ALK1 (and ALK3) signaling. They are under phase I/II trials as clinical treatments for HHT. Preclinical studies are investigating the beneficial effects of anti-ANGPT2 antibodies (LC-10) and PI3-kinase inhibitors (wortmannin or LY294002). As shown on this cartoon, all these treatments aim at restoring the balance between the BMP9 pathway and the VEGF/ANGPT2 pathways in order to re-establish vascular quiescence

Although current treatments succeed pretty well at reducing recurrent epistaxis, there is still a need for « magic bullets » allowing to revert telangiectasias and AVMs into a normal vasculature and to definitely cure the disease. Two distinct drug repositioning strategies have been developed recently to achieve this goal. One is to reposition anti-angiogenic drugs used in cancer therapy (anti-VEGF antibody, tyrosine-kinase inhibitors) for counter-balancing the pro-angiogenic process activated in HHT. The other is to blindly screen drug libraries using an HHT mechanism-based cellular assay. Fig. 3 depicts the distinct sites of action of such identified drugs whereas Table 1 summarizes the recent case reports about HHT patients treated by these candidate drugs.

**Table 1:** New treatments in HHT, case reports and case series

Ref.	Treatment	n (HHT Type)	Sex/ Age	Symptoms	Treatment indication	Treatment	Efficacy of treatment
[44]	Tacrolimus	1 (HHT2)	M 51	Epistaxis GI bleeding	Epistaxis	Dose unknown	↓ Epistaxis
[45]	Pazopanib	1 (HHT2)	M 61	Epistaxis Anemia	Epistaxis	50 mg/d during 1 month then: 100 mg/d	↓Epistaxis
[46]	Pazopanib	7 (3 HHT1, 3 HHT2, 1 JP/HHT)		Anemia Epistaxis	Anemia, OR, severe epistaxis with iron deficiency	50 mg /d during 12 weeks	↓epistaxis duration ↗ Hb ↗ SF-36
[47]	Nintedanib	1 (HHT2)	M 70	Epistaxis Telangiectasias	Pulmonary fibrosis	300 mg/d	↓Epistaxis and telangiectasias
[48]	Sunitinib	1 (?)	M 68	Epistaxis Multiple metastases	Oncology	37.5 mg/d	↓ epistaxis frequency and intensity ↓facial telangiectasia
[49]	Buparlisib	1 (HHT2)	F 49	Epistaxis	Oncology	100 mg/d	↓ frequency of epistaxis

### Anti-angiogenic therapies using Bevacizumab

Since 2012, following two promising case reports [50, 51], anti-angiogenic treatment using Bevacizumab, a humanized monoclonal antibody that selectively binds to and neutralizes the biologic activity of human VEGF, was tested on HHT patients in several clinical trials.

In the first phase II trial, Bevacizumab was administered intravenously to HHT patients (22 HHT2, 2HHT1, 1 JP-HHT) complicated by severe liver and cardiac impairments. The trial highlighted the efficacy of this treatment, not only on the liver lesions, as shown by a decrease in the cardiac hyperflow secondary to hepatic vessel malformations, but also on nosebleeds, which were considerably reduced, thereby strongly improving the quality of life of the patients [52]. No severe adverse events related to Bevacizumab have been observed.

Since then, many case reports showing dramatic improvement of HHT bleedings (epistaxis and digestive bleedings) after bevacizumab treatment have been published [53–57] and this treatment is now considered in HHT patients with refractory GI bleeding [58].

The first randomized phase III clinical trial to study bevacizumab efficiency and safety is now ongoing (NCT 03227263).

### Anti-angiogenic therapies using tyrosine-kinase inhibitors

Among tyrosine kinase inhibitors are some anti-angiogenic molecules which could also target the VEGF signaling pathway, in a similar way to Bevacizumab. These chemical compounds are available orally and could therefore overcome the constraints of intravenous injections of Bevacizumab.

Several of them have been tested on isolated HHT patients as summarized in Table 1.

The potential therapeutic effects of four anti-angiogenic tyrosine-kinase inhibitors in the development of adult-onset AVMs in a murine model of HHT was evaluated [59]. The conclusion was that Sorafenib and a Pazopanib analogue (GW771806) significantly improved hemoglobin level and gastro-intestinal bleeding whereas they were not effective in preventing wound-induced skin AVMs.

The tyrosine kinase inhibitor Nintedanib, which targets the platelet-derived growth factor, fibroblast growth factor and vascular endothelial growth factor receptors, has been used in one HHT2 patient following the diagnosis of Interstitial Pulmonary Fibrosis [47] with encouraging results. His Epistaxis Severity Score significantly decreased.In France, we are implementing a multicenter, randomized, drug versus placebo study to evaluate efficacy of Nintedanib treatment per os on epistaxis duration in HHT patients with moderate to severe epistaxis (NCT03954782).

Pazopanib, another tyrosine kinase inhibitor, has been tested at a dose of 50 mg/day in 3 HHT1, 3 HHT2 and 1 JP-HHT patients by Faughnan et al. [46] and showed promising results in treatment of HHT-related bleeding (NCT02204371). Unfortunately, this industry-driven study was stopped. However, two other studies are planned in North America using this drug (NCT03850730 and NCT03850964).

### Anti-ANGPT2 antibodies and PI3 kinase inhibitors

It is hypothesized that neutralization of angiogenic factors (VEGF and others) re-equilibrates the balance between pro- and anti-angiogenic factors that is altered by the inactivation of the pro-quiescence BMP9 pathway (Fig. 3). As angiopoietin-2 (ANGPT2) is a potent angiogenic factor acting through the tyrosine-kinase receptor Tie2, anti-ANGPT2 antibodies have been tested in preclinical models of HHT consisting of Smad4-KO mice [38]. They were shown to alleviate AVM formation and to normalize blood vessel diameter. Similarly, as activation of PI3-Kinase is downstream of both VEGF and ANGPT2, PI3-Kinase inhibitors have been tested and have proven efficacy in preclinical HHT models such as *Alk1*^+/−^ mice or mice treated with neutralizing anti-BMP9/BMP10 antibodies [40, 42]. They are interesting candidates which remain to be tested in clinical trials on HHT patients. Interestingly, an older case report had reported that treatment of one HHT2 patient with Buparlisib (a PI3-Kinase inhibitor) reduced the frequency of his epistaxes [49]. However, there might be major safety and tolerability challenges for their chronic use due to the reported adverse effects of the first generation of PI3 Kinase inhibitors [60].

#### Tacrolimus

Now that the genetic studies have pointed to a precise signaling pathway, one can envision to develop targeted therapies or to reposition existing drugs that would target the BMP9/ALK1/ENG/SMAD pathway. Since the disease occurs in heterozygous patients and results from haploinsuffficiency, a rather simplistic strategy would consist in reactivating this pathway in endothelial cells in order to recover the signaling level of homozygous cells. In other terms, if a drug could double the rate of Smad 1/5/9 phosphorylation by wild type ALK1, then the endothelial cells of HHT patients bearing 50% of wild type ALK1 receptors should behave as normal biallelic cells and the disease might reverse.

Finding the best readout to screen molecules potentiating the BMP9 pathway is a complex task since the signaling pathway is quite direct, with BMP9 receptors phosphorylating the Smad1/5/9 transcription factors that will, in turn, translocate to the nucleus and trigger the transcriptional response. In order to detect drugs that can act on most numerous steps, the best method appears to measure the transcriptional activation of a BMP9-responsive gene. For the sake of easiness and efficacy, the most commonly used reporter gene encodes firefly luciferase driven by an artificial promoter comprising tandem repeats of the BMP-response element of the Id1 (Inhibitor of differentiation-1) gene promoter [61].

Zilberberg et al. have generated a C2C12 myoblastic cell line stably expressing this construct (C2C12BRA) which provides a highly sensitive assay to measure BMP activities [62]. This cell line has been used by two groups to reposition FDA-approved drugs for pulmonary arterial hypertension (PAH) and HHT. Spiekerkoetter et al. and Ruiz et al. respectively screened 3756 FDA-approved drugs (NIH-CC, LOPAC, Biomol ICCB KnownBioactives, Microsource spectrum, Biomol FDA-approved drug libraries) and 700 FDA-approved drugs (NIH Clinical, NCCS) on C2C12BRA cells [63, 64]. The group of Spiekerkoetter screened for agents able to stimulate luciferase expression and activity in the absence or the presence of low concentrations of exogenous ligand (250pM BMP4 = EC_20_). The best three hits were immunosuppressant agents: FK506 (Tacrolimus), ascomycin and rapamycin (Sirolimus). The group of Ruiz performed a similar screen in C2C12BRA cells in the presence of BMP9 (0.5 ng/ml = EC_50_). Interestingly, the most potent activating drug that they found was again Tacrolimus. Together, these two high-throughput screens clearly identified Tacrolimus as a potent activator of the BMP9-ALK1-BMPR2-Smad1/5/9 signaling cascade. How Tacrolimus activates this pathway is still not completely understood. Tacrolimus (FK506) can bind to FKBP12 (FK-506-binding protein-12), a protein known to interact with the TGF-ß/BMP family type I receptors on their glycine-serine-rich phosphorylation domains and to repress the receptors’ kinase activity in the absence of their ligands. Tacrolimus was shown indeed to displace FKBP12 from ALK1, ALK2 as well as ALK3 and to stimulate their kinase activity, explaining how it potentiates both the BMP9 response (through ALK1) and the BMP4 response (through ALK3) [64]. Alternatively, Tacrolimus was also reported to stimulate endoglin and ALK-1 expression by endothelial cells [65]. Tacrolimus has been tested in several mouse models for HHT. It was found to decrease the number of retinal arteriovenous malformations induced by BMP9/10-immunodepletion in mice (HHT model) [63]. These preclinical works support that Tacrolimus repurposing has therapeutic potential in HHT.

Interestingly, a case report of a patient suffering from both HHT2 and PAH was recently published that showed that treatment with oral low-dose tacrolimus improved his HHT-associated epistaxis but did not attenuate PAH progression [44].

All these observations prompted us to set up a recent clinical trial to evaluate nasal topical administration of tacrolimus in HHT patients. This phase II multicenter, randomized study (NCT03152019) was carried out in double blind in order to evaluate the efficacy of this nasal ointment. This ointment is administered for 6 weeks to patients with HHT complicated by nosebleeds and the final readout is the duration of nosebleeds 6 weeks after the end of the treatment. Results are encouraging (presented at the 13th HHT International Conference, Rio Grande, Puerto Rico, USA, 2019) and are under current analysis.

#### Sirolimus

Very recently, Ruiz et al. reported that the combination of Sirolimus (rapamycin) and Nintedanib reversed retinal AVMs in BMP9/BMP10-immunoblocked mice and prevented gastrointestinal bleeding and anemia in adult Alk1-inducible KO mice [66]. Sirolimus binds FKBP12 and inhibits mTOR (mammalian Target Of Rapamycin), which is downstream of PI3K and AKT, and this could be another mechanism by which this drug targets this pathway. Indeed, the beneficial effects observed in these preclinical models were associated with a correction of the overactivation of both VEGFR2 and mTOR.

When Sirolimus was given following liver transplantation to a patient with HHT who had multiple arteriovenous malformations, internal and external telangiectasia, epistaxis, and anemia disappeared, suggesting that the mechanism of action of sirolimus involved partial correction of endoglin and ALK1 haploinsufficiency [67]. Interestingly, it was also observed that, in HHT2 patients with hepatic AVMs and high-output cardiac failure that undergo a liver transplantation and receive immunosuppressive treatments with tacrolimus or sirolimus, their epistaxes improved dramatically [68] and their mucosal bleedings stopped. Their hemoglobin levels normalized and cutaneous and gastrointestinal telangiectases disappeared.

More recently, Sirolimus was reported to be efficient and safe for the treatment of blue rubber bleb nevus syndrome, a rare multifocal venous malformation syndrome involving predominantly the skin and gastrointestinal tract [69], as well as for other venous and lymphaticovenous malformations [70].

#### Other drug screening approach

Another ongoing approach uses phenotypic screening of siRNA-silenced endothelial cells. The company Recursion Pharmaceuticals is currently using ALK1-silenced endothelial cells in order to identify drugs that reverse the ALK1 siRNA-induced phenotype (Gibson CC. Oral presentation at the 13th HHT international conference. Rio Grande, Puerto Rico, USA, unpublished). They have already validated a similar strategy for the treatment of cerebral cavernous malformations [71].

## Conclusion

Since the discovery some 25 years ago that *ENG* or *ACVRL1* gene mutations cause HHT [8, 9], significant progress has been made in the comprehension of the biological mechanisms of this pathology.

It is puzzling however, that no etiological therapeutic treatment targeting the mutated components of the BMP9/10-ALK1-Smad1/5/9 signaling pathway has been developed so far. This review focuses on the possible repositioning of existing drugs that either correct the angiogenic defects of HHT patients (Bevacizumab, tyrosine kinase inhibitors, PI3 Kinase inhibitors) or reactivate the altered BMP9/10 signaling pathway (Tacrolimus, Sirolimus). It is now reasonably predictable that these mechanism-driven drugs will soon enter clinical assays and enlarge the therapeutic arsenal available for the treatment of HHT patients.

## Data Availability

Not applicable.

## Abbreviations

5′-UTR: 5′-untranslated region
ACTRIIA/B: Activin receptor type IIA/B
*ACVRL1* gene: Activin receptor-like kinase 1 gene
AKT: AKT serine/threonine protein-kinase
ANGPT2: Angiopoietin 2
AVM: Arteriovenous malformation
BMPRII: Bone morphogenetic protein receptor II
BMPx: Bone morphogenetic protein X
EMA: European Medicines Agency
*ENG* gene: Endoglin gene; ALKx: activin receptor-like kinase X
FDA: Food and Drug Administration
FKBP12: FK-506-binding protein-12
*GDF2* gene: Growth and differentiation factor 2/bone morphogenetic protein 9 gene
GI bleeding: Gastro-intestinal bleeding
GS box: Glycine- and serine-rich box
HHT: Hereditary hemorrhagic telangiectasia
JP: Juvenile polyposis
mTOR: Mammalian target of rapamycin
PAH: Pulmonary arterial hypertension
PI3K: Phosphatidylinositol-4,5 bisphosphate 3-kinase
PTEN: Phosphatase and tensin homolog
VEGF: Vascular endothelial growth factor
VEGFR1/2: Vascular endothelial growth factor 1/2

## References

[1] Govani FS, Shovlin CL (2009). Hereditary haemorrhagic telangiectasia: a clinical and scientific review. Eur J Hum Genet.

[2] Guttmacher AE, Marchuk DA, White RI (1995). Hereditary hemorrhagic telangiectasia. N Engl J Med.

[3] Lesca G, Genin E, Blachier C, Olivieri C, Coulet F, Brunet G (2008). Hereditary hemorrhagic telangiectasia: evidence for regional founder effects of ACVRL1 mutations in French and Italian patients. Eur J Hum Genet.

[4] Plauchu H, de Chadarevian JP, Bideau A, Robert JM (1989). Age-related clinical profile of hereditary hemorrhagic telangiectasia in an epidemiologically recruited population. Am J Med Genet.

[5] Dupuis-Girod S, Bailly S, Plauchu H (2010). Hereditary hemorrhagic telangiectasia: from molecular biology to patient care. J Thromb Haemost.

[6] Buscarini E, Plauchu H, Garcia Tsao G, White RI, Sabba C, Miller F (2006). Liver involvement in hereditary hemorrhagic telangiectasia: consensus recommendations. Liver Int.

[7] Kjeldsen AD, Kjeldsen J (2000). Gastrointestinal bleeding in patients with hereditary hemorrhagic telangiectasia. Am J Gastroenterol.

[8] Johnson DW, Berg JN, Baldwin MA, Gallione CJ, Marondel I, Yoon SJ (1996). Mutations in the activin receptor-like kinase 1 gene in hereditary haemorrhagic telangiectasia type 2. Nat Genet.

[9] McAllister KA, Grogg KM, Johnson DW, Gallione CJ, Baldwin MA, Jackson CE (1994). Endoglin, a TGF-beta binding protein of endothelial cells, is the gene for hereditary haemorrhagic telangiectasia type 1. Nat Genet.

[10] Niessen K, Zhang G, Ridgway JB, Chen H, Yan M (2010). ALK1 signaling regulates early postnatal lymphatic vessel development. Blood..

[11] Seki T, Yun J, Oh SP (2003). Arterial endothelium-specific activin receptor-like kinase 1 expression suggests its role in arterialization and vascular remodeling. Circ Res.

[12] Gougos A, Letarte M (1990). Primary structure of endoglin, an RGD-containing glycoprotein of human endothelial cells. J Biol Chem.

[13] Saito T, Bokhove M, Croci R, Zamora-Caballero S, Han L, Letarte M (2017). Structural basis of the human Endoglin-BMP9 interaction: insights into BMP signaling and HHT1. Cell Rep.

[14] Gallione CJ, Repetto GM, Legius E, Rustgi AK, Schelley SL, Tejpar S (2004). A combined syndrome of juvenile polyposis and hereditary haemorrhagic telangiectasia associated with mutations in MADH4 (SMAD4). Lancet..

[15] Gallione CJ, Richards JA, Letteboer TG, Rushlow D, Prigoda NL, Leedom TP (2006). SMAD4 mutations found in unselected HHT patients. J Med Genet.

[16] Lesca G, Burnichon N, Raux G, Tosi M, Pinson S, Marion MJ (2006). Distribution of ENG and ACVRL1 (ALK1) mutations in French HHT patients. Hum Mutat.

[17] Hernandez F, Huether R, Carter L, Johnston T, Thompson J, Gossage JR (2015). Mutations in RASA1 and GDF2 identified in patients with clinical features of hereditary hemorrhagic telangiectasia. Hum Genome Var.

[18] Wooderchak-Donahue WL, McDonald J, O'Fallon B, Upton PD, Li W, Roman BL (2013). BMP9 mutations cause a vascular-anomaly syndrome with phenotypic overlap with hereditary hemorrhagic telangiectasia. Am J Hum Genet.

[19] David L, Mallet C, Mazerbourg S, Feige JJ, Bailly S (2007). Identification of BMP9 and BMP10 as functional activators of the orphan activin receptor-like kinase 1 (ALK1) in endothelial cells. Blood..

[20] Scharpfenecker M, van Dinther M, Liu Z, van Bezooijen RL, Zhao Q, Pukac L (2007). BMP-9 signals via ALK1 and inhibits bFGF-induced endothelial cell proliferation and VEGF-stimulated angiogenesis. J Cell Sci.

[21] Tillet E, Ouarne M, Desroches-Castan A, Mallet C, Subileau M, Didier R (2018). A heterodimer formed by bone morphogenetic protein 9 (BMP9) and BMP10 provides most BMP biological activity in plasma. J Biol Chem.

[22] Garcia de Vinuesa A, Abdelilah-Seyfried S, Knaus P, Zwijsen A, Bailly S (2016). BMP signaling in vascular biology and dysfunction. Cytokine Growth Factor Rev.

[23] Goumans MJ, Zwijsen A, Ten Dijke P, Bailly S (2017). Bone morphogenetic proteins in vascular homeostasis and disease. Cold Spring Harb Perspect Biol.

[24] Tillet E, Bailly S (2014). Emerging roles of BMP9 and BMP10 in hereditary hemorrhagic telangiectasia. Front Genet.

[25] Damjanovich K, Langa C, Blanco FJ, McDonald J, Botella LM, Bernabeu C (2011). 5'UTR mutations of ENG cause hereditary hemorrhagic telangiectasia. Orphanet J Rare Dis.

[26] Ricard N, Bidart M, Mallet C, Lesca G, Giraud S, Prudent R (2010). Functional analysis of the BMP9 response of ALK1 mutants from HHT2 patients: a diagnostic tool for novel ACVRL1 mutations. Blood..

[27] Mallet C, Lamribet K, Giraud S, Dupuis-Girod S, Feige JJ, Bailly S (2015). Functional analysis of endoglin mutations from hereditary hemorrhagic telangiectasia type 1 patients reveals different mechanisms for endoglin loss of function. Hum Mol Genet.

[28] Gallione C, Aylsworth AS, Beis J, Berk T, Bernhardt B, Clark RD (2010). Overlapping spectra of SMAD4 mutations in juvenile polyposis (JP) and JP-HHT syndrome. Am J Med Genet A.

[29] McDonald J, Wooderchak-Donahue W, VanSant WC, Whitehead K, Stevenson DA, Bayrak-Toydemir P (2015). Hereditary hemorrhagic telangiectasia: genetics and molecular diagnostics in a new era. Front Genet.

[30] Park SO, Wankhede M, Lee YJ, Choi EJ, Fliess N, Choe SW (2009). Real-time imaging of de novo arteriovenous malformation in a mouse model of hereditary hemorrhagic telangiectasia. J Clin Invest.

[31] Hao Q, Su H, Marchuk DA, Rola R, Wang Y, Liu W (2008). Increased tissue perfusion promotes capillary dysplasia in the ALK1-deficient mouse brain following VEGF stimulation. Am J Physiol Heart Circ Physiol.

[32] Snellings D, Gallione C, Clark D, Vozoris N, Faughnan M, Marchuk D. Somatic mutations in vascular malformations of hereditary hemorrhagic telangiectasia results in biallelic loss of ENG or ACVRL1. Am J Hum Genet. 2019;105:894–906. 10.1016/j.ajhg.2019.09.010 PMC6848992 31630786

[33] Bidart M, Ricard N, Levet S, Samson M, Mallet C, David L (2012). BMP9 is produced by hepatocytes and circulates mainly in an active mature form complexed to its prodomain. Cell Mol Life Sci.

[34] David L, Mallet C, Keramidas M, Lamande N, Gasc JM, Dupuis-Girod S (2008). Bone morphogenetic protein-9 is a circulating vascular quiescence factor. Circ Res.

[35] Wood JH, Guo J, Morrell NW, Li W (2019). Advances in the molecular regulation of endothelial BMP9 signalling complexes and implications for cardiovascular disease. Biochem Soc Trans.

[36] Larrivee B, Prahst C, Gordon E, del Toro R, Mathivet T, Duarte A (2012). ALK1 signaling inhibits angiogenesis by cooperating with the notch pathway. Dev Cell.

[37] Thalgott JH, Dos-Santos-Luis D, Hosman AE, Martin S, Lamande N, Bracquart D (2018). Decreased expression of vascular endothelial growth factor receptor 1 contributes to the pathogenesis of hereditary hemorrhagic telangiectasia type 2. Circulation..

[38] Crist AM, Zhou X, Garai J, Lee AR, Thoele J, Ullmer C (2019). Angiopoietin-2 inhibition rescues Arteriovenous malformation in a Smad4 hereditary hemorrhagic telangiectasia mouse model. Circulation.

[39] Ruiz S, Zhao H, Chandakkar P, Chatterjee PK, Papoin J, Blanc L (2016). A mouse model of hereditary hemorrhagic telangiectasia generated by transmammary-delivered immunoblocking of BMP9 and BMP10. Sci Rep.

[40] Alsina-Sanchis E, Garcia-Ibanez Y, Figueiredo AM, Riera-Domingo C, Figueras A, Matias-Guiu X (2018). ALK1 loss results in vascular hyperplasia in mice and humans through PI3K activation. Arterioscler Thromb Vasc Biol.

[41] Iriarte A, Figueras A, Cerda P, Mora JM, Jucgla A, Penin R (2019). PI3K (phosphatidylinositol 3-kinase) activation and endothelial cell proliferation in patients with hemorrhagic hereditary telangiectasia type 1. Cells..

[42] Ola R, Dubrac A, Han J, Zhang F, Fang JS, Larrivee B (2016). PI3 kinase inhibition improves vascular malformations in mouse models of hereditary haemorrhagic telangiectasia. Nat Commun.

[43] Ola R, Kunzel SH, Zhang F, Genet G, Chakraborty R, Pibouin-Fragner L (2018). SMAD4 prevents flow induced Arteriovenous malformations by inhibiting casein kinase 2. Circulation.

[44] Sommer N, Droege F, Gamen KE, Geisthoff U, Gall H, Tello K (2019). Treatment with low-dose tacrolimus inhibits bleeding complications in a patient with hereditary hemorrhagic telangiectasia and pulmonary arterial hypertension. Pulm Circ.

[45] Parambil JG, Woodard TD, Koc ON (2018). Pazopanib effective for bevacizumab-unresponsive epistaxis in hereditary hemorrhagic telangiectasia. Laryngoscope..

[46] Faughnan ME, Gossage JR, Chakinala MM, Oh SP, Kasthuri R, Hughes CCW (2019). Pazopanib may reduce bleeding in hereditary hemorrhagic telangiectasia. Angiogenesis..

[47] Kovacs-Sipos E, Holzmann D, Scherer T, Soyka MB (2017). Nintedanib as a novel treatment option in hereditary haemorrhagic telangiectasia. BMJ Case Rep.

[48] Droege F, Thangavelu K, Lang S, Geisthoff U (2016). Improvement in hereditary hemorrhagic telangiectasia after treatment with the multi-kinase inhibitor Sunitinib. Ann Hematol.

[49] Geisthoff UW, Nguyen HL, Hess D (2014). Improvement in hereditary hemorrhagic telangiectasia after treatment with the phosphoinositide 3-kinase inhibitor BKM120. Ann Hematol.

[50] Flieger D, Hainke S, Fischbach W (2006). Dramatic improvement in hereditary hemorrhagic telangiectasia after treatment with the vascular endothelial growth factor (VEGF) antagonist bevacizumab. Ann Hematol.

[51] Mitchell A, Adams LA, MacQuillan G, Tibballs J, vanden Driesen R, Delriviere L (2008). Bevacizumab reverses need for liver transplantation in hereditary hemorrhagic telangiectasia. Liver Transpl.

[52] Dupuis-Girod S, Ginon I, Saurin JC, Marion D, Guillot E, Decullier E (2012). Bevacizumab in patients with hereditary hemorrhagic telangiectasia and severe hepatic vascular malformations and high cardiac output. JAMA..

[53] Buscarini E, Botella LM, Geisthoff U, Kjeldsen AD, Mager HJ, Pagella F (2019). Safety of thalidomide and bevacizumab in patients with hereditary hemorrhagic telangiectasia. Orphanet J Rare Dis..

[54] Epperla N, Hocking W (2015). Blessing for the bleeder: bevacizumab in hereditary hemorrhagic telangiectasia. Clin Med Res.

[55] Fleagle JM, Bobba RK, Kardinal CG, Freter CE (2012). Iron deficiency anemia related to hereditary hemorrhagic telangiectasia: response to treatment with bevacizumab. Am J Med Sci.

[56] Follner S, Ibe M, Schreiber J (2012). Bevacizumab treatment in hereditary hemorrhagic teleangiectasia. Eur J Clin Pharmacol.

[57] Lupu A, Stefanescu C, Treton X, Attar A, Corcos O, Bouhnik Y (2013). Bevacizumab as rescue treatment for severe recurrent gastrointestinal bleeding in hereditary hemorrhagic telangiectasia. J Clin Gastroenterol.

[58] Iyer VN, Apala DR, Pannu BS, Kotecha A, Brinjikji W, Leise MD (2018). Intravenous Bevacizumab for refractory hereditary hemorrhagic telangiectasia-related epistaxis and gastrointestinal bleeding. Mayo Clin Proc.

[59] Kim YH, Kim MJ, Choe SW, Sprecher D, Lee YJ, S PO (2017). Selective effects of oral antiangiogenic tyrosine kinase inhibitors on an animal model of hereditary hemorrhagic telangiectasia. J Thromb Haemost.

[60] Esposito Angela, Viale Giulia, Curigliano Giuseppe (2019). Safety, Tolerability, and Management of Toxic Effects of Phosphatidylinositol 3-Kinase Inhibitor Treatment in Patients With Cancer. JAMA Oncology.

[61] Korchynskyi O, ten Dijke P (2002). Identification and functional characterization of distinct critically important bone morphogenetic protein-specific response elements in the Id1 promoter. J Biol Chem.

[62] Zilberberg L, ten Dijke P, Sakai LY, Rifkin DB (2007). A rapid and sensitive bioassay to measure bone morphogenetic protein activity. BMC Cell Biol.

[63] Ruiz S, Chandakkar P, Zhao H, Papoin J, Chatterjee PK, Christen E (2017). Tacrolimus rescues the signaling and gene expression signature of endothelial ALK1 loss-of-function and improves HHT vascular pathology. Hum Mol Genet.

[64] Spiekerkoetter E, Tian X, Cai J, Hopper RK, Sudheendra D, Li CG (2013). FK506 activates BMPR2, rescues endothelial dysfunction, and reverses pulmonary hypertension. J Clin Invest.

[65] Albinana V, Sanz-Rodriguez F, Recio-Poveda L, Bernabeu C, Botella LM (2011). Immunosuppressor FK506 increases endoglin and activin receptor-like kinase 1 expression and modulates transforming growth factor-beta1 signaling in endothelial cells. Mol Pharmacol.

[66] Ruiz S, Zhao H, Chandakkar P, Papoin J, Choi H, Nomura-Kitabayashi A, et al. Correcting Smad1/5/8, mTOR, and VEGFR2 treats pathology in hereditary hemorrhagic telangiectasia models. J Clin Invest. 2019; 10.1172/JCI127425. 10.1172/JCI127425 PMC6994128 31689244

[67] Skaro AI, Marotta PJ, McAlister VC (2006). Regression of cutaneous and gastrointestinal telangiectasia with sirolimus and aspirin in a patient with hereditary hemorrhagic telangiectasia. Ann Intern Med.

[68] Dupuis-Girod S, Chesnais AL, Ginon I, Dumortier J, Saurin JC, Finet G (2010). Long-term outcome of patients with hereditary hemorrhagic telangiectasia and severe hepatic involvement after orthotopic liver transplantation: a single-center study. Liver Transpl.

[69] Salloum R, Fox CE, Alvarez-Allende CR, Hammill AM, Dasgupta R, Dickie BH (2016). Response of blue rubber bleb nevus syndrome to Sirolimus treatment. Pediatr Blood Cancer.

[70] Yesil S, Tanyildiz HG, Bozkurt C, Cakmakci E, Sahin G (2016). Single-center experience with sirolimus therapy for vascular malformations. Pediatr Hematol Oncol.

[71] Gibson CC, Zhu W, Davis CT, Bowman-Kirigin JA, Chan AC, Ling J (2015). Strategy for identifying repurposed drugs for the treatment of cerebral cavernous malformation. Circulation.

